# Supplementary figures and images for: Genetic interactions of G-quadruplexes in humans
Source: eLife. 2019 Jul 9;8:e46793. doi: 10.7554/eLife.46793 (PMC6615864; doi:10.7554/eLife.46793)

**A**

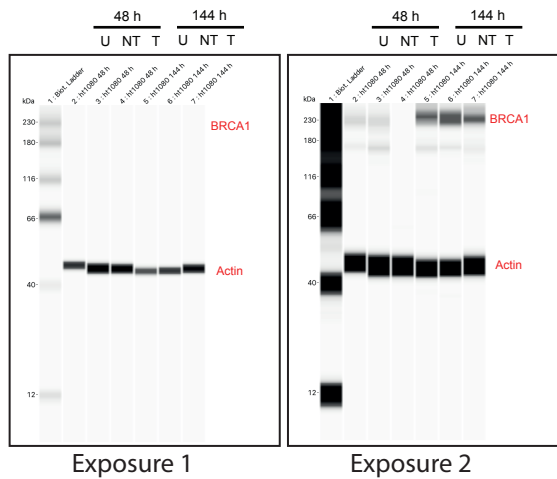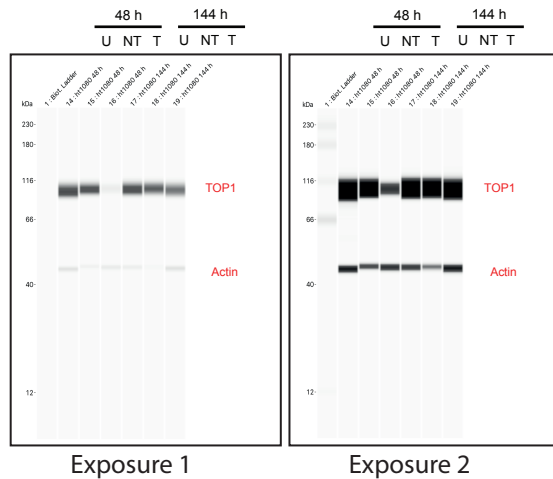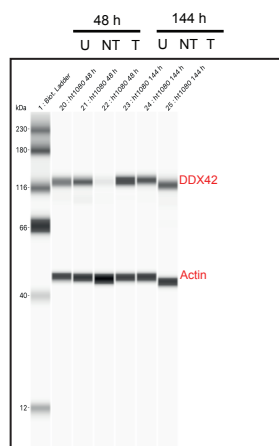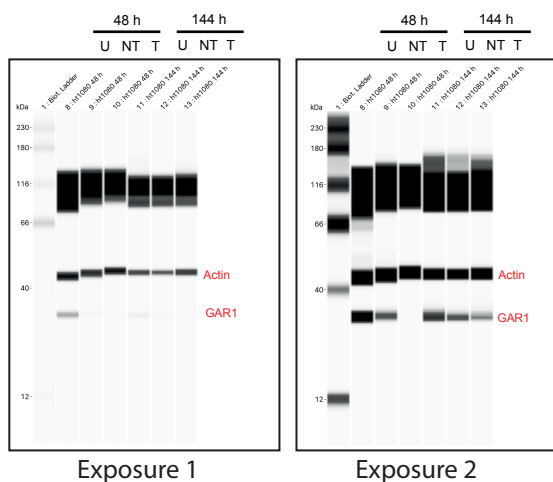

**B**

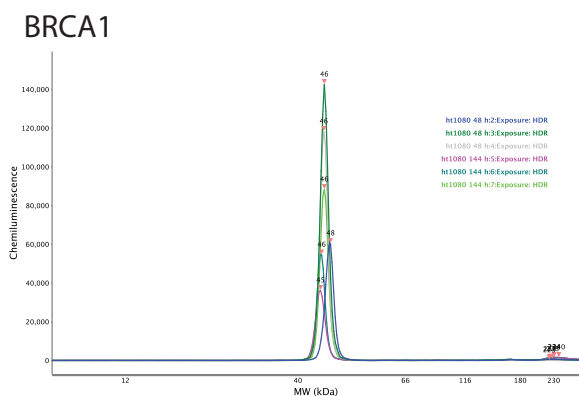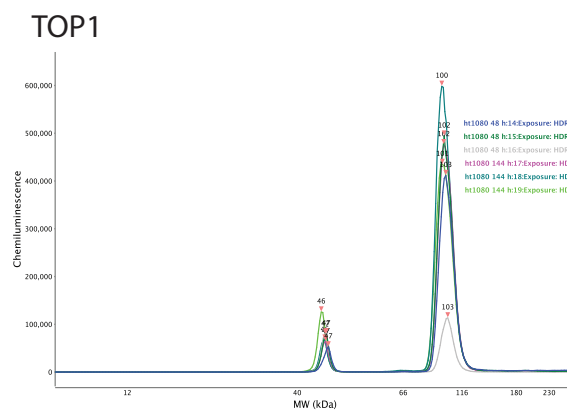

Supplement: Figure 8—source data 1. — (A) Full length western blots and (B) capillary traces obtained from Compass Software (Simple Western) for results shown in Figure 8A. [file elife-46793-fig8-data1.pdf]

**A**

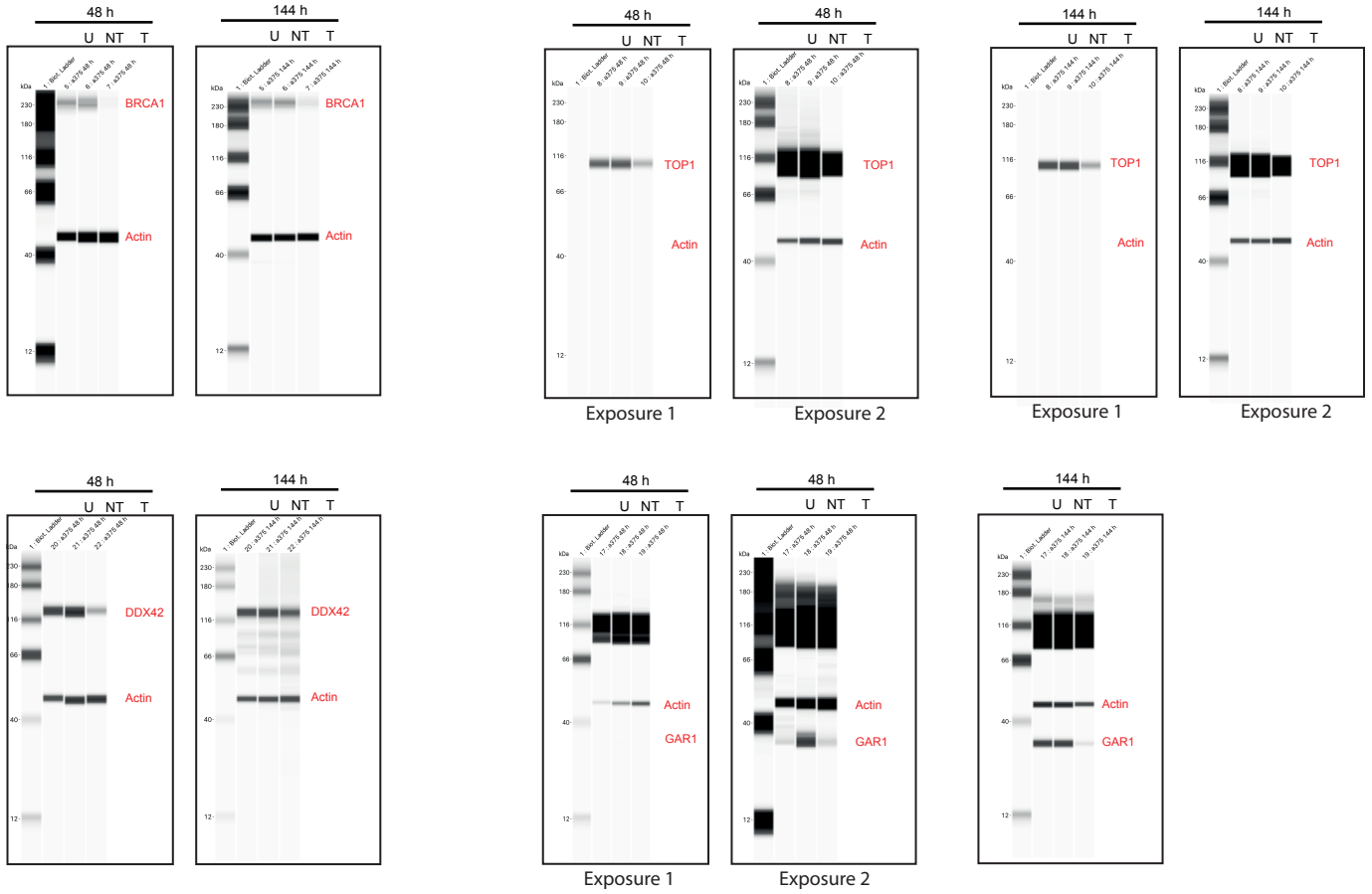

**B**

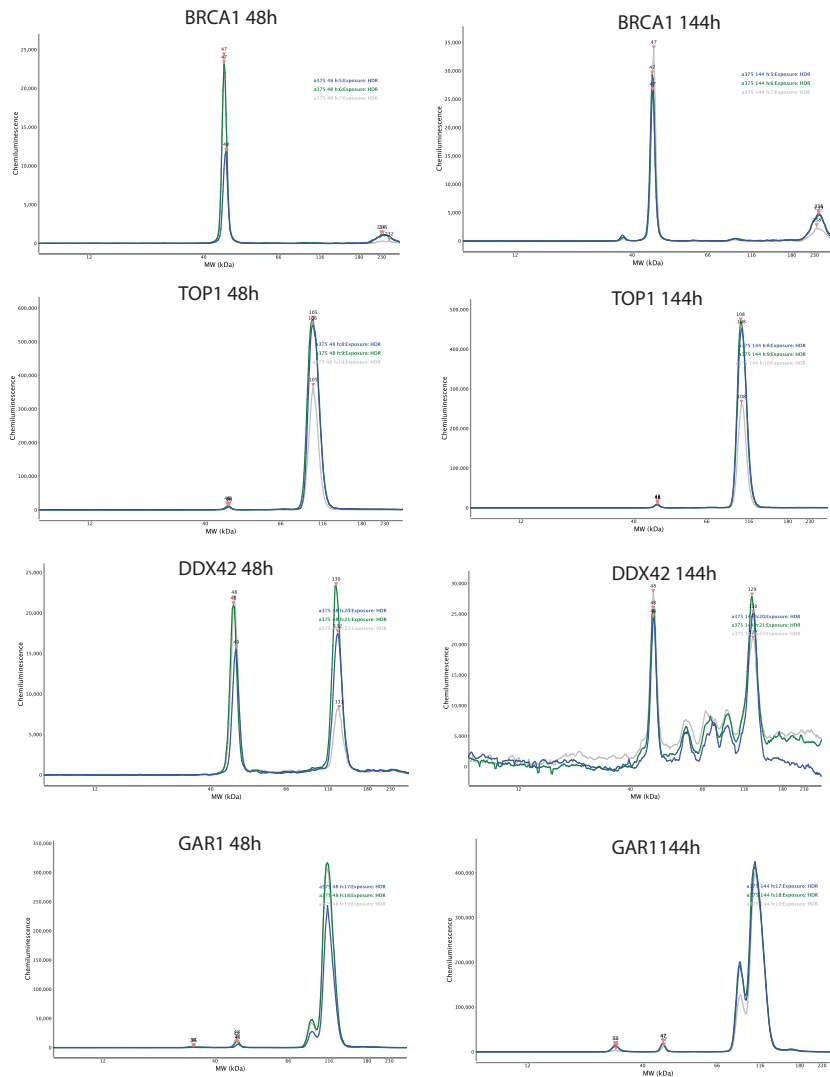

Supplement: Figure 8—figure supplement 3—source data 1. — (A) Full length western blots and (B) capillary traces obtained from Compass Software (Simple Western) for results shown in Figure 8—figure supplement 3A. [file elife-46793-fig8-figsupp3-data1.pdf]

A

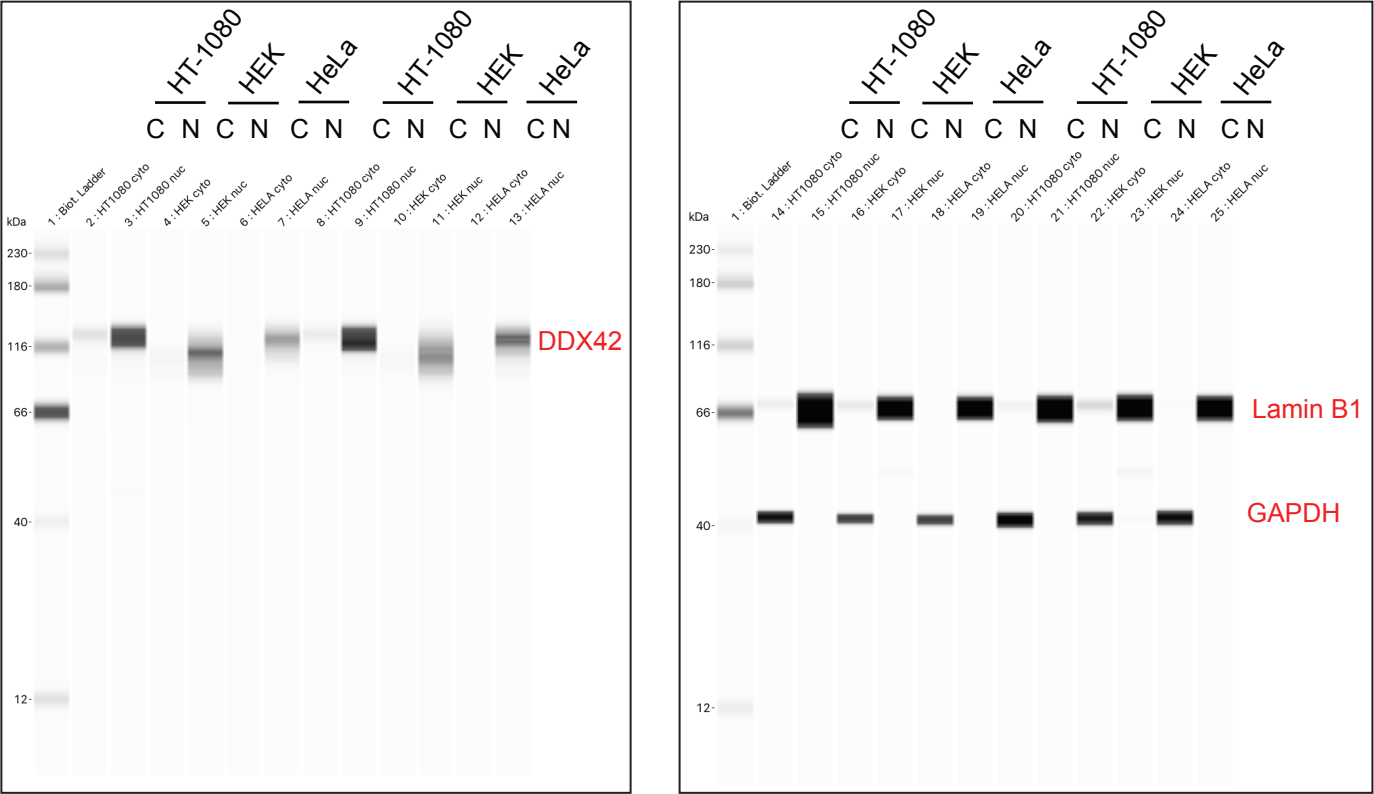

B

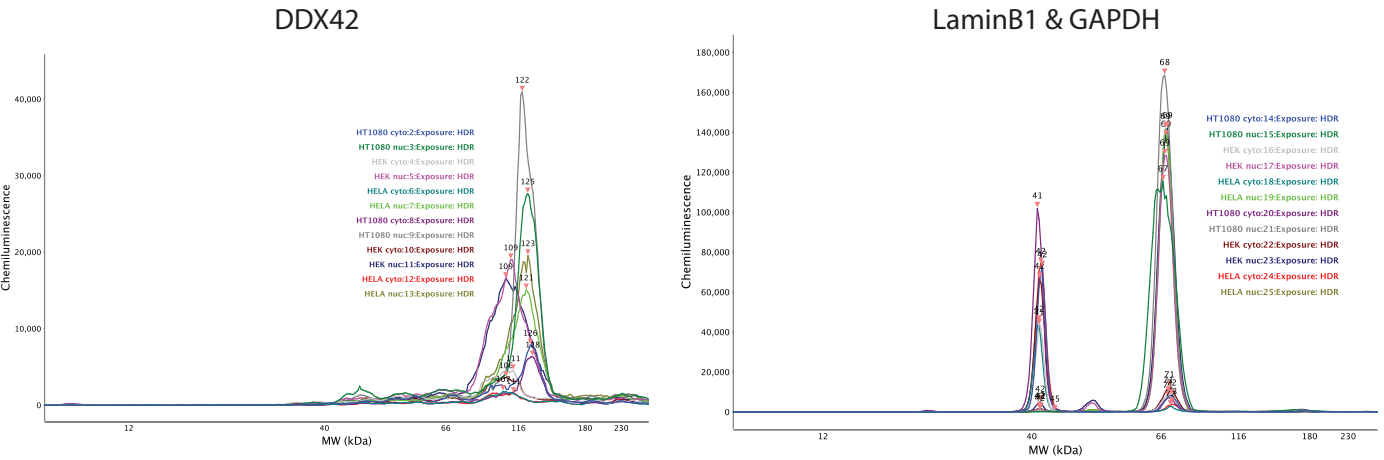

Supplement: Figure 10—source data 1. — (A) Full-length western blots and (B) capillary traces obtained from Compass Software (Simple Western) for results shown in Figure 10A. [file elife-46793-fig10-data1.pdf]
