# Supplementary material for: Genetic interactions of G-quadruplexes in humans
Source: eLife. 2019 Jul 9;8:e46793. doi: 10.7554/eLife.46793 (PMC6615864; doi:10.7554/eLife.46793)
Supplement: Transparent reporting form [file elife-46793-transrepform.docx]

***eLife’s* transparent reporting form**

We encourage authors to provide detailed information *within their submission* to facilitate the interpretation and replication of experiments. Authors can upload supporting documentation to indicate the use of appropriate reporting guidelines for health-related research (see [EQUATOR Network](http://www.equator-network.org/%20)), life science research (see the [BioSharing Information Resource](https://biosharing.org/)), or the [ARRIVE guidelines](http://www.plosbiology.org/article/info:doi/10.1371/journal.pbio.1000412) for reporting work involving animal research. Where applicable, authors should refer to any relevant reporting standards documents in this form.

If you have any questions, please consult our Journal Policies and/or contact us: [editorial@elifesciences.org](mailto:editorial@elifesciences.org).

**Sample-size estimation**

- You should state whether an appropriate sample size was computed when the study was being designed
- You should state the statistical method of sample size computation and any required assumptions
- If no explicit power analysis was used, you should describe how you decided what sample (replicate) size (number) to use

Please outline where this information can be found within the submission (e.g., sections or figure legends), or explain why this information doesn’t apply to your submission:

No explicit power calculation was used. All RNAi screens used 1000 and 800 cells for each shRNA for A375 and HT1080 cell lines, respectively, as indicated in Methods and Figure 2D and Figure 6A Legends, to ensure maximal potential for uncovering phenotypic effects from each shRNA hairpin tested. Duration of the RNAi screens was predetermined from the results of the pilot RNAi screen, as described in the text, Figure 2- figure supplement 1 and Methods.

**Replicates**

- You should report how often each experiment was performed
- You should include a definition of biological versus technical replication
- The data obtained should be provided and sufficient information should be provided to indicate the number of independent biological and/or technical replicates
- If you encountered any outliers, you should describe how these were handled
- Criteria for exclusion/inclusion of data should be clearly stated
- High-throughput sequence data should be uploaded before submission, with a private link for reviewers provided (these are available from both GEO and ArrayExpress)

Please outline where this information can be found within the submission (e.g., sections or figure legends), or explain why this information doesn’t apply to your submission:

Information on the type and number of replicates can be found in the Figure Legends and methods throughout the text. All RNAi screens were performed in three biological replicates as described in the Methods. Access to the RNAi screen sequencing data and computer code can be found at the following link: <https://github.com/sblab-bioinformatics/GWscreen_G4sensitivity> as described in Methods Section “Sequencing, read processing, alignment and counting of shRNAs”). For each shRNA library, shRNA hairpins bearing less than 0.5 counts-per-million were excluded from analysis.

**Statistical reporting**

- Statistical analysis methods should be described and justified
- Raw data should be presented in figures whenever informative to do so (typically when N per group is less than 10)
- For each experiment, you should identify the statistical tests used, exact values of N, definitions of center, methods of multiple test correction, and dispersion and precision measures (e.g., mean, median, SD, SEM, confidence intervals; and, for the major substantive results, a measure of effect size (e.g., Pearson's r, Cohen's d)
- Report exact p-values wherever possible alongside the summary statistics and 95% confidence intervals. These should be reported for all key questions and not only when the p-value is less than 0.05.

Please outline where this information can be found within the submission (e.g., sections or figure legends), or explain why this information doesn’t apply to your submission:

Statistical methods used to analyse data can be found in the Methods and Figure Legends. Bonferroni adjustment to correct for multiple hypothesis testing was used for KEGG and GO term enrichments (Figures 4, 6D-E and 7C and Figure 6-figure supplement1F-G). Mean and standard deviation is reported for Figures 8B-F and 10C-G, Figure 2-figure supplement 1A, Figure 6-figure supplement 1A, Figure 7-figure supplement 1A, Figure 8-figure supplement 1 & 2, and Figure 8-figure supplement 3B-J. For the analysis of RNAi sequencing libraries, the negative binomial generalize linear model was used to model treatment counts and false discovery rates were estimated using Benjamini-Hochberg method for each shRNA hairpin. Fisher’s exact test was used to calculate fold enrichment of discovered gene sensitisers that are COSMIC cancer genes.

(For large datasets, or papers with a very large number of statistical tests, you may upload a single table file with tests, Ns, etc., with reference to sections in the manuscript.)

**Group allocation**

- Indicate how samples were allocated into experimental groups (in the case of clinical studies, please specify allocation to treatment method); if randomization was used, please also state if restricted randomization was applied
- Indicate if masking was used during group allocation, data collection and/or data analysis

Please outline where this information can be found within the submission (e.g., sections or figure legends), or explain why this information doesn’t apply to your submission:

N/A

**Additional data files (“source data”)**

- We encourage you to upload relevant additional data files, such as numerical data that are represented as a graph in a figure, or as a summary table
- Where provided, these should be in the most useful format, and they can be uploaded as “Source data” files linked to a main figure or table
- Include model definition files including the full list of parameters used
- Include code used for data analysis (e.g., R, MatLab)
- Avoid stating that data files are “available upon request”

Please indicate the figures or tables for which source data files have been provided:

Custom analysis pipeline, scripts and link to ArrayExpress for the genome-wide, focused and pilot RNAi screens can be found at the following link: <https://github.com/sblab-bioinformatics/GWscreen_G4sensitivity>, which can also be found in the Methods Section “Sequencing, read processing, alignment and counting of shRNAs”.

Source data for all reported Venn Diagrams created from shRNA screen data can be found in Supplementary Files 1 and 2. Raw data for drug synergy experiments (Figure 9) is reported in Figure 9-figure supplement 1. Full length blots and capillary images for western blots reported in Figure 8A, 10A and Figure 8-figure supplement 3A can be found in Figure 8 - Source data 1, Figure 10 - Source File 1 and Figure 8-figure supplement 3- Source data 1, respectively.
